# Ultrashort Version of the Utrecht Work Engagement Scale (UWES-3): A Psychometric Assessment

**DOI:** 10.3390/ijerph19020890

**Published:** 2022-01-14

**Authors:** César Merino-Soto, Milagros Lozano-Huamán, Sadith Lima-Mendoza, Gustavo Calderón de la Cruz, Arturo Juárez-García, Filiberto Toledano-Toledano

**Affiliations:** 1 Instituto de Investigación en Psicología, Universidad de San Martin de Porres, Lima 15024, Peru; sikayax@yahoo.com.ar (C.M.-S.); gcalderond@usmp.pe (G.C.d.l.C.); 2 Escuela de Psicología, Universidad de San Ignacio de Loyola, Lima 15024, Peru; milagroslozanoha@gmail.com (M.L.-H.); sadhit-1-07@hotmail.com (S.L.-M.); 3 Centro de Investigación Transdisciplinar en Psicología, Universidad Autónoma del Estado de Morelos, Cuernavaca 62350, Mexico; arturojuarezg@hotmail.com; 4 Unidad de Investigación en Medicina Basada en Evidencias, Hospital Infantil de México Federico Gómez, Mexico City 06720, Mexico; 5 Unidad de Investigación Sociomédica, Instituto Nacional de Rehabilitación Luis Guillermo Ibarra Ibarra, Mexico City 14389, Mexico

**Keywords:** engagement, validity, job satisfaction, accidents at work, stress

## Abstract

The objective was to determine the validity of the UWES-3, an ultrashort measure of work engagement lacking evidence in Hispanic populations. In total, 200 workers with heterogeneous positions and careers from Metropolitan Lima were enrolled via nonprobabilistic sampling. The UWES-3 and measures of external variables (work accidents, stress overload, and others) were used. Data were collected through a web platform. Items were analysed, nonparametric response theory methods (Mokken scale analysis and Ramsay curves) were applied to the items, and ordinal and linear regression were used to determine the relationships with external variables. The items had statistically similar distributional properties and monotonic associations with external variables but with fewer functional response options. The UWES-3 complied with the monotonic homogeneity model and invariant ordering of items; the scaling of the items, score (greater than 0.80), and reliability (0.94) were high. With the effects of age and sex controlled, the UWES-3 significantly predicted minor accidents at work and job satisfaction and revealed effects of stress overload and perceived efficacy. The theoretical implications of the UWES-3 as a brief unidimensional measure integrating the three original dimensions of the instrument and the practical implications of its use for research and professional practice are discussed.

## 1. Introduction

From positive psychology, one of the models of positive mental states that has gained recognition and diffusion explores the connection between the energy that activates workers, their wellbeing, and their effective performance. Engagement is the representative construct in this type of study, and it is characterized by the combined and synergistic presence of vigour (high levels of energy, mental resistance, will and persistence in the face of difficulties at work), dedication (strong involvement and identification with work) and absorption (concentration and enjoyment at work and difficulty detaching from it) [1,2,3].

Engagement has been shown to be antagonistic to burnout syndrome, that is, emotional exhaustion vs. vigour and cynicism vs. dedication [2,4,5]; however, [6] argue that given the complexity of the results on the relationship between burnout and engagement, its original dimensions must be maintained by adhering to the greater psychological connections of its processes to clarify these relationships. In the job demands–resources model (JDR; [7,8,9]), engagement is explained as an experience of positive stress originating from favourable work conditions that is increased by labour resources (e.g., autonomy at work), personal factors (e.g., self-efficacy [10] and challenging job demands [11]) and decreased when the work environment is highly demanding [12]. The JDR model also explains that the consequences of engagement favourably influence workers’ motivation [2,13], performance [14], and wellbeing [15].

Among the measurement instruments used for the evaluation of engagement, the Utrecht Work Engagement Scale (UWES) predominates [1,16]. This scale has been translated into 31 languages, and it integrates the dimensions of vigour, dedication and absorption. It initially contained 17 items (UWES-17; [2]); later, a short version with nine items was developed (UWES-9; [1]), preserving the three dimensions of the original. The psychometric properties of each of the versions has been measured, making them useful in the context of development studies. However, recently, an ultrabrief version composed of three items (UWES-3; [3]) was developed. The item selection was verified via face validity through a rational and consensual process based on the validation results obtained from the UWES-17 and UWES-9, thus reducing the dependence on sample variations and idiosyncrasy and corresponding to the data collection processes.

To obtain other evidence of validity, the UWES-3 was applied in the countries of Spain, Finland, Japan, the Netherlands, and Belgium [3]. In this way, its metric equivalence with the UWES-9 was demonstrated, and then these two versions were compared following the JDR model, corroborating their associations with burnout, work demands (e.g., work overload), work resources (e.g., feedback), personal resources (e.g., general self-efficacy) and consequential organizational factors (e.g., organizational commitment) [3].

The main contribution of the UWES-3 is that it is an ultrabrief measurement instrument that can measure the psychosocial wellbeing of workers within the framework of evaluating psychosocial factors at work (demands and work resources) and work performance and its consequences for worker health and labour organization. Given that this type of evaluation usually involves a large number of items and requires a significant amount of time to complete, participants may have negative reactions to both pencil-and-paper and online versions [17]. In this sense, obtaining an ultrabrief measure of engagement evaluation is highly valuable in contexts where a screening measurement, monthly monitoring, or incorporation into a long survey is required.

However, it is not known whether the results on the psychometric properties of the UWES-3 are generalizable in studies that include Spanish-speaking participants. For Peru, the instrumental studies published with the UWES seem to provide only initial and provisional evidence due to their scarcity, design, or scope; for example, there is only one Peruvian psychometric study on the internal structure of the UWES-15 and UWES-9 with schoolteachers [18]. In other Latin American contexts, no study using the new ultrashort version has been conducted. Although engagement and burnout seem to be accepted as predominantly emic constructs, that is, constructs with external generalizability and validity in various cultures [19], the shift in the conceptualization of a multidimensional measure to its conceptualization as one-dimensional measure requires an evaluation of its rationality and its replicability in other contexts. Therefore, additional studies of the UWES-3 are needed to ensure the continuity of its use and cross-cultural interpretation and to avoid extending it to other contexts of use for which it has not been validated, that is, to establish its measurement validity [20,21].

The present study aims to present evidence of the validity of the UWES-3 in a sample of Spanish-speaking workers (Peruvians). The validity framework follows the approach of modern validity theory [22], according to which construct validity is viewed as a unitary concept composed of various sources of evidence that support the interpretation and use of instrument scores. This evaluation focuses on two sources of validity evidence: the characteristics of the internal structure (dimensionality, measurement invariance, and score reliability [22]) and the relationship with other constructs and criteria [22], specifically through the associations of engagement with perceived efficacy, stress overload, minor occupational accidents, and work satisfaction. Among the many variables eligible for inclusion in a validity study, the present research selects job satisfaction, stress overload, and perceived efficacy because of the consistent relationships between these constructs and engagement. The literature supporting this relationship is integrated into the arguments defending the hypotheses. Therefore, regarding this last source of validity evidence, we propose several hypotheses:

**Hypothesis** **1** **(H1)**. *The linear association between perceived efficacy and engagement is positive. Previous evidence has verified that self-efficacy is an individual attribute and is influential for the emergence of engagement [23,24], and in general, in the JDR model, self-efficacy is a personal resource that contributes to an increase in engagement [3]. Therefore, it is proposed that the variability of the UWES-3 is positively influenced by the variability of the antecedent perceived efficacy.*

**Hypothesis** **2** **(H2)**. *The association between stress overload and engagement is a second-degree polynomial. Stress overload is understood as a definitional process of the experience of stress [25], and from the transactional conception [26], stress emerges from the interactions between subjects and their environments. The ineffectiveness in this interaction leads to an increase in negative experiences of stress. Evidence has shown that stress, then, at high levels, is a negative condition that increases the deterioration of health [27] and is a precursor to the triggering of burnout and other problems, while at efficiently managed levels, it can increase engagement [28]. This suggests a nonlinear or curvilinear relationship; however, based on the high levels of stress in other Peruvian samples [29], high stress scores are expected in this sample and, therefore, it is expected that the variability of said scores of the overload measure of stress is negatively associated with the UWES-3 scores in the context in which a curvilinear polynomial model better represents this association.*

**Hypothesis** **3** **(H3)**. *The linear association between engagement and the estimated number of minor work accidents is negative. Regarding engagement and the accident rate, there is evidence that when workers are engaged, the perceived job security climate is linked to a decrease in labour demands, and in general, there is a decrease in incidents of occupational accidents, as well as a lower frequency of behaviours that expose them to accidents [30]. In this sense, engagement can act as an antecedent of accident rate [31], and we propose that the variability of the UWES-3 negatively influences the self-reported number of minor accidents at work.*

**Hypothesis** **4** **(H4)**. *The linear association between engagement and work satisfaction is positive. Both constructs are conceptually and empirically independent [32], but the associations with engagement are consistent and tend to be higher at small magnitudes of correlation [32,33,34]. Theoretically, it is understood that engaged workers will respond better to work tasks, value their work more and, as a consequence, feel a greater degree of satisfaction. Therefore, we propose that the variability of the UWES-3 positively influences job satisfaction scores.*

## 2. Materials and Methods

### 2.1. Participants

The reference population comprised Peruvian workers of various positions and professions with a formal contract in for-profit or nonprofit institutions. The eligibility of the participants consisted of being of legal age (18 years) and actively employed at the time of the survey. The exclusion criteria were having a high rate of missing responses and not having consented to participate by means of the informed consent form.

### 2.2. Instruments

The *UWES-3* [3] is an abbreviated measure of engagement evaluation, sharing the same model as its previous versions (i.e., UWES-15 and UWES-9). It is composed of three items: feeling energy (vigour), enthusiasm (dedication), and immersion (absorption). Its response options are ordinally scaled from 0 (never) to 6 (always/every day). The internal consistency reliability (α) in the original study [3] ranged from 0.77 to 0.85.

#### 2.2.1. Work Satisfaction

The work satisfaction instrument was adapted from Kunin’s [35] face scale and measured the overall job satisfaction perceived by the worker. The prompt was as follows: “Consider all aspects of your job and mark the face that best describes how you feel about your job in general.” The scaling of the response options was expressed with symbolic smiley face labels, with five equally spaced faces [36], in which the arc representing the mouth varied from concave position to convex, with the rest of the representation remaining constant. Although there is evidence that suggests that the straight line in the mouth representing a neutral expression may function differentially between men and women [37], the scaling expressed by the smiley faces [38], especially with five faces, works well to represent one-dimensional and equidistant measurement objects [36].

#### 2.2.2. Stress Overload

The Stress Overload Scale-Brief (SOS-S; Amirkhan [25]) assesses the perception of stress overload with 10 items, expressed in two dimensions: personal vulnerability (5 items, ω = 0.958, bootstrap-95% CI = 0.904, 0.946) and event load (5 items, ω = 0.927, bootstrap-95% CI = 0.903, 0.945). For the present study, the items were translated by a panel of five Latino researchers with English language proficiency, using a consensus method. In the present study, the items were adjusted to a one-dimensional model (ULSMW-χ^2^ = 9.10, *p* > 0.10, CFI = 1.00, SRMR = 0.029, WRMR = 0.327) and a two-dimensional model (ULSMW-χ^2^ = 13.89, *p* > 0.10, CFI = 1.00, SRMR = 0.036, WRMR = 0.404), and due to the statistical similarity of both settings and the high correlation between both dimensions (r = 0.942), the one-dimensional model was used (ω = 0.956, bootstrap-95% CI = 0.944, 0.965). The unidimensionality obtained was similar to that found in other studies [39,40].

#### 2.2.3. General Coping Effectiveness

One item was used to measure the perceived global efficacy to face adversities, experienced as difficulties, and represented the perceived effect of using strategies or resources to face difficulties or stressors. It was phrased as “I can cope with the difficulties (problems) that come my way” and had five options (strongly disagree to strongly agree). This measure was influenced by the development of short efficient and valid measures applied in a work context [41].

#### 2.2.4. Minor Accidents at Work

A single-item self-reported measure of occupational injuries was developed as a proxy for the frequency of minor incidents (“In the last 12 months, have you had minor accidents at work (minor cuts or bruises) that did not require medical attention?”, with the response options: none, rarely, occasionally, frequently, very frequently). Due to their relationship with the perception of a safe environment [42,43] and their efficiency in identifying key areas of accidents, obtaining prevalence, monitoring occupational safety, and contrasting information provided by employees, these types of single-item measures are usually applied in national epidemiological surveys or independent studies [44,45,46,47].

### 2.3. Procedures

Data collection. The survey was prepared on a free web platform, with the sections appearing in the following order: brief presentation of the study, informed consent form (information about the voluntary nature of participation, the anonymity and confidentiality of the responses, the absence of tangible benefits, the availability of results or inquiries about the study to the authors, and the freedom to withdraw at any time), demographic questions, and the content of the instruments (each preceded by specific instructions). The generated link was sent en masse to the participants selected from the agendas and social networks of the study authors in an invitation to participate. This invitation was brief and explained the purpose and importance of the research, as well as the possibility of sending the authors an image of the completed survey, so that it could be progressively reviewed to ensure that each test had been answered in its entirety. The data collection was performed between November 2020 and February 2021. This research followed all the precepts of the Helsinki Declaration on Research in Humans [48].

### 2.4. Ethical Considerations

This study is a part of the research project HIM/2015/017/SSA.1207 “Effects of mindfulness training on psychological distress and quality of life of the family caregiver,” which was approved on 16 December 2014, by the Research, Ethics, and Biosafety Commissions of the Hospital Infantil de México Federico Gómez, National Institute of Health, in Mexico City. While conducting this study, the ethical rules and considerations for research with humans currently enforced in Mexico [49] and those outlined by the American Psychological Association [50] were followed. All family caregivers were informed of the objectives and scope of the research and their rights according to the Declaration of Helsinki [48]. The caregivers who agreed to participate in the study signed an informed consent letter. Participation in this study was voluntary and did not involve payment.

### 2.5. Statistical Analysis

Items were first analysed regarding their distributional properties, and second, regarding their linear associations (through the Pearson product-moment correlation; Rcompanion package [51] for the SOS-S and number of accidents at work); finally, they were assessed regarding the association with satisfaction associative labour and perceived self-efficacy, and the linear correlations obtained were corrected for granularity [52] due to the ordinal expression of the assumed latent distribution of the constructs (the R psychmeta package was used: Dahlke and Wiernik [53]).

To examine the psychometric properties of the set of items, a nonparametric approach was applied to evaluate the fundamental properties of the scores and to obtain weighted scores that maximized the validity of the observed score. Regarding the first, because the number of items in the UWES-3 is lower than the number used in general evaluative practice [54] and because parametric modelling is very short [55,56], Mokken scale analysis (MSA; Mokken [57], an effective item response theory nonparametric approach (IRTNP)) was applied to solve the precursor psychometric properties of parametric modelling [57,58,59]. Two main models were investigated: (a) the latent monotonicity model (MHM) and (b) invariant item ordering (IIO), both with item scalability. The MHM and IIO models were evaluated with the crit index to estimate the impact of violations on these models [60], which consists of the weighted sum of several fulfilled conditions (e.g., number of violations to the model). Although there was a discrepancy for the crit cut-off point (crit > 40, Stochl et al. [61]; crit > 80, Molenaar and Sijtsma [60]), in the present study, a conservative criterion was used (crit > 80). Scalability was estimated with the coefficient H between items (Hij) for each item (Hj) and with the total score. Reliability within the MSA modelling was estimated with the rho coefficient of Molenaar–Sijtsma (rhoMS; Sijtsma and Molenaar [62]), and their confidence intervals were generated by bootstrap simulation (a copy of the R code can be requested from the main author). The MSA approach was implemented with the R mokken package [63].

To examine the relationship with other constructs, the second nonparametric IRT procedure (IRTNP) was the estimation of maximum likelihood scores (pML) of the participants from the modelling of Ramsay curves [64,65]. This procedure consists of regressions of smoothed kernel curves (in the present study, the kernel function was of the Gaussian type) generated to estimate the weight of the monotonic relationship between the item, and the ranking derived from the observed score, and they were then added for the estimation of the pML of the characteristic curves of the items and other diagnoses of the functioning of the items and their response options [65]. Compared with complex parametric models, this nonparametric method is particularly efficient for properly identifying the item–construct relationship under various data conditions [66,67,68]. This method was implemented with the KernSmoothIRT program [69]. With these optimal scores, ordinal regression was applied (ordinal R program; Christensen [70]), as was a second-degree linear and polynomial regression (R stats program; and lm.beta, Behrendt [71]) with global evaluation of their statistical assumptions (program R gvlma; Pena and Slate [72]).

## 3. Results

### 3.1. Participant Sample

The participating sample consisted of 200 workers (men, 101, 50.2%), with 134 unmarried (55.7%), 64 married or cohabiting (31.8%), and 3 without data. They resided in various districts of Metropolitan Lima. Regarding the degree of education, 39 had a basic education (19.4%), 117 had a technical education (58.2%), and 45 had a higher education (25.4%). According to the International Standard Classification of Occupations (ISCO-08; [73]), the workers were distributed in the following positions: directors and managers (3; 1.5%); scientific and intellectual professionals (58, 28.9%); technicians and mid-level professionals (65; 32.3%); administrative support staff (35, 17.4%); services and vendors of shops and markets (9; 4.5%); officials, operators, artisans and mechanical arts and other trades (26; 12.9%); plant and machine operators and assemblers (4; 2.0%); and elementary occupations (1; 0.5%). The careers of the participants were classified as follows: health sciences (10; 3.2%); basic sciences (1; 0.5%); engineering (25; 10.6%); economic sciences and management (65; 34.6%); humanities, legal and social sciences (41; 20.7%); does not belong/does not apply (59; 29.8%); and no data (3; 0.5%).

### 3.2. Item Analysis

The descriptive, structural and correlational results are presented in Table 1. The interitem correlations and scalability of the items were high (> 0.84 in both), suggesting high congruence in the responses. The association with external variables was similar for each item of the UWES-3 for work satisfaction (*r* mean = 0.56), stress overload (SOS-S, *r* mean = 0.59), general coping effectiveness (GCE, *r* mean = 0.34), and the number of minor work accidents (*r* mean = −0.23), and the direction in all had theoretical convergence. The structure of the response options did not appear to be efficient for the first two options because the response frequency was predominantly less than 10. For subsequent analyses, these two options were merged. The items did not show multivariate normality (Hense–Zirkler test = 10.91, *p* < 0.001) or univariate normality (AD test with *p* < 0.01; see Table 1).

### 3.3. Dimensionality

**Figure 1:**
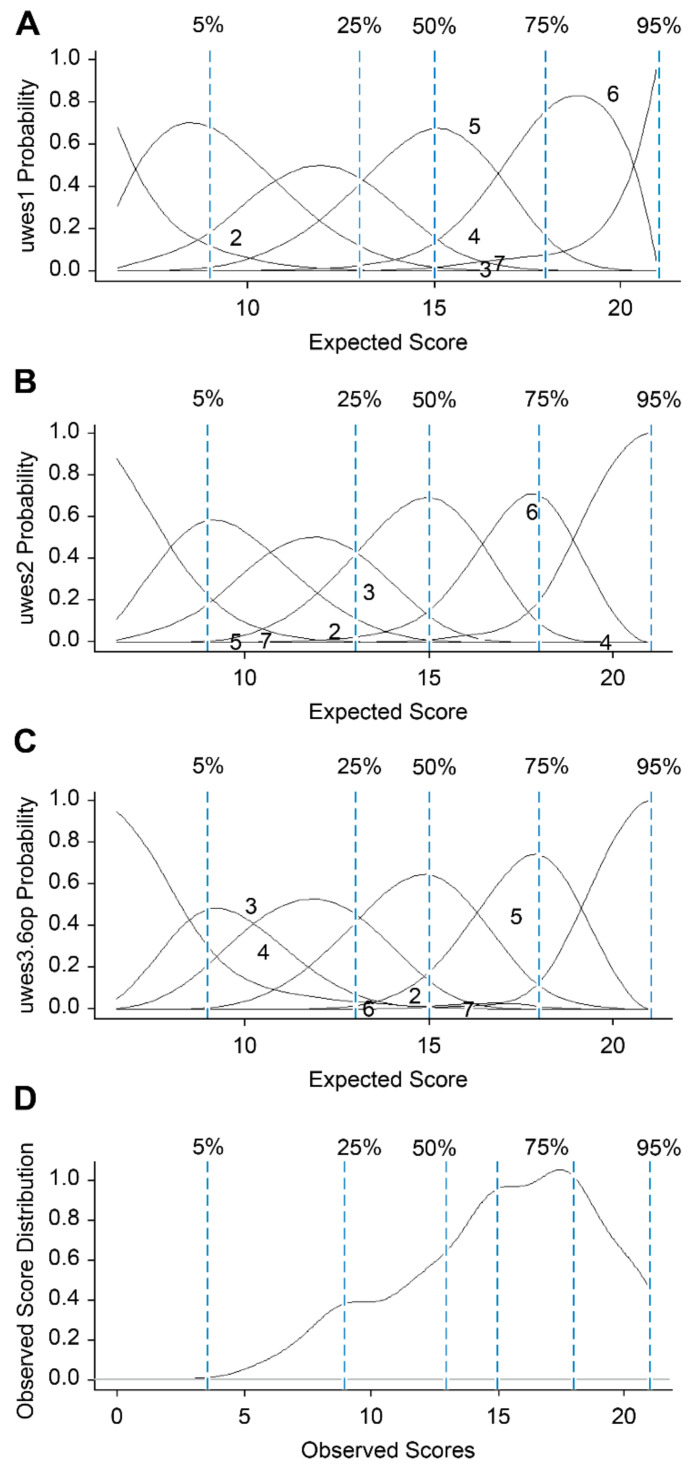
Item option characteristic curves and kernel distribution of the UWES-3 total score. (**A**–**C**): items 1, 2, and 3, respectively. (**D**): UWES-3 observed total score.

Table 2 shows the results of the MSA modelling, showing that the latent homogeneity and invariant ordering models of the items were effectively completed. The scalability of the items and the total score were high and very similar. Overall, the UWES-3 showed a satisfactory Mokken score. Figure 1 shows the characteristic curves of the response options estimated with the Ramsay curves, in which good differentiation was observed between each response option, with a sufficient distance between them, an ordered sequence of options, and a strong global similarity of their distributions along the expected score. In the lower graph (Figure 1), the density of the distribution of the score is higher over the median, which also corresponds to the density of the distribution of the characteristic curves of the response options.

### 3.4. Reliability

A rhoMS coefficient = 0.94 was obtained, with a 95% confidence interval (BCA, 1000 replications) between 0.92 and 0.95; sampled and populationally, this magnitude of reliability was high.

### 3.5. Relationship with Other Variables

The assumptions for applying linear regression were fulfilled in the model that included the SOS-S predictor (χ^2^ global = 15.02, *p* < 0.01) with respect to symmetry (χ^2^ = 6.44, *p* < 0.01) and the link function (χ^2^ = 7.22, *p* < 0.01), but not in the model with the IUFD predictor (χ^2^ global = 7.11, *p* > 0.10), so statistical significance was estimated with bootstrap simulation (1000 simulated samples). Using the UWES-3 as a criterion, stress overload (SOS-S) and general coping effectiveness (IUFD) were statistically significant (F > 7.0) after controlling for sex and age, and were in the theoretical direction expected (negative and positive, respectively); see Table 3. The quadratic effect of the SOS-S was also statistically significant, and its beta^2^ coefficient was higher than that of the linear beta. On the other hand, in the ordinal regression (criteria: minor work accidents and work satisfaction; Table 3), the UWES-3 maintained a statistically significant association (likelihood ratio test > 10.0; gl: 7) while controlling for the effect of age and sex. In all models, sex and age had no effect on linear or ordinal modelling.

## 4. Discussion

This is one of the first studies to verify the validity of an ultrabrief measure of engagement, the UWES-3 [3]. All validity evidence was satisfactory, which indicates the potential of the instrument to briefly (with three items) represent a conceptually integrated three-dimensional construct that is traditionally measured with 17 or 9 items, with a limited number of items. Regarding dimensionality, the results showed that the UWES-3 has a Mokken score that indicates that it can effectively differentiate workers in terms of their level of engagement. This finding was supported by the differentiation achieved in the response options but not in the first two options. These two options were not functional because they attracted a low-frequency response. A consequent implication is that the ordinal scaling of the items can be modified in five or six options, but this suggestion must be contextualized by the sample size of the study. Thus, for applied purposes, better response efficiency can be achieved with five options. In this line of internal structure results, the reliability was very high and suggests applied uses to describe not only groups of workers, but also individual workers; therefore, the score achieves a high level of intraindividual replicability. This level of reliability far exceeds the minimum cut-off point (alpha > 0.70; Lance et al. [74]) commonly established in organizational/industrial psychology.

Regarding the relationship with other variables, the results verify that the UWES-3 score is associated with a decrease in minor accidents at work and an increase in job satisfaction, while it decreases as a consequence of stress overload in a curvilinear manner and increases in the presence of perceived efficacy to face difficulties. According to the DR model and to intuitive observation, this pattern confirms the validity of the UWES-3 and additionally corroborates the theory and empirical findings in relation to minor occupational accidents [30,31] and the rest of the constructs analysed [28,32,33,34]. Overall, the UWES-3 is sensitive to individual processes and consequences of work–worker interaction.

We must point out that the theory and empirical evidence on stress generally suggest that its association with performance or engagement criteria would be better specified with the modelling of the curvilinear association. This indicates that an increase in, for example, engagement is conditioned nonlinearly by the amount of perceived stress. Therefore, linear modelling, as applied in the study, would not properly capture the magnitude of a theoretically expected curvilinear association. However, in the data, it was possible to obtain a statistically significant estimate of negative orientation (of the beta coefficient, once the effects of sex and age were controlled for), indicating that the negative linear relationship and the curvilinear effect were effective.

A methodological implication of the study is the application of nonparametric procedures to verify each of the proposed goals and to maximize the representation of the direct score with respect to its construct. Although a simple sum of the items is the usual method to represent the construct in a self-report measure such as the UWES-3 for research purposes and to overcome the limitations introduced by the sample size and the small number of items, the maximum likelihood scores derived from the Ramsay [64,65] method represent an appropriate route. Due to the sample size and the scale model (three items), the application of other models, such as a structural equation model, risks obtaining invalid conclusions about the fit in relation to the consequences of having few degrees of freedom in the model and its effect on discrepancy between their population and sample values, high overestimation bias in the adjustment indices, and decisions [75].

In addition to the practical advantages of using a brief instrument for evaluating engagement, the theoretical implications of the results point to the one-dimensional suitability of a construct that represents three characteristics traditionally treated as distinct (vigour, absorption, and dedication). In the present study, the integration of work engagement into a single construct is confirmed, the mechanism of which constitutes the set of psychological effects presented simultaneously as a single synergistic process and probably not of temporal dependence between them. This is relevant not only because of the parsimonious use of interpretation from the sum of the total score and not independent scores, but also because of the application of the UWES-3 to health promotion programs from the positive psychology framework.

Finally, the limitations of the study are due to the sample (i.e., sample size and population representativeness), the study design (the cross-sectional design used does not allow causal statements about the relationship between the UWE-3 and the external variables used), and the heterogeneity of the groups of workers. Additionally, some measures were not previously validated for the Peruvian worker population (or even in the Latin American context), which may raise legitimate doubts about the validity of their constructs. However, this same study serves as a source of initial evidence to validate these instruments as well, particularly due to the theoretical coherence found in the results. Finally, another limitation was that measurement invariance was not evaluated with structural equation modelling (SEM). As a counterpart to these limitations, the present study (a) did not intend to draw definitive but rather initial but robust conclusions; (b) the cross-sectional design was effective for the psychometric hypotheses created; and (c) the validity of the complementary measures used can be inferred from the results of the present results (the statistically significant associations and their magnitude validate each instrument used in both directions) and from the accumulated research on them, that is, on the effective measurement of general job satisfaction through pictorial representations [76,77,78], perceived coping efficacy [41], and self-reported minor occupational accidents [42,43,44,46,47]. As a final counterpart related to the absent measurement invariance, Mokken analysis on item invariant ordering is an accepted nonparametric approach for the assessment of measurement invariance [79].

## 5. Conclusions

It is concluded that the shortened version of the UWES shows favourable psychometric evidence for its use in applied and research contexts, and therefore, it could be useful when the costs of extensive or prolonged evaluations are an important limitation for the researcher or professional. The three items of the UWES-3 originally come from three different but strongly associated theoretical dimensions of the UWES, from which a shortened one-dimensional version was constructed. The responses to the items showed similar distributional and descriptive properties among them. The number of efficient response options (five) is less than that in the original measure (seven), and its use in small samples may require restructuring into five response options. The items show high and similar scalability values and satisfy the model of monotonic homogeneity and invariant ordering of the items; they also show strong discriminative capacity, as well as a relationship with the total score. For the external variables (job satisfaction, stress overload, coping with difficulties, and number of minor accidents at work), the items are associated in a similar way and show a consistent theoretical direction.

The score is highly consistent and can generate reproducible results with high reliability, and for external variables (job satisfaction, stress overload, coping with difficulties, and number of minor accidents at work), the association is replicated for the items with these criteria. In summary, the ultrashort version of a known measure of work engagement, the UWES-3, showed strong psychometric properties. Given its brevity, it is an efficient measure of a construct linked to wellbeing at work and labour productivity.

## Figures and Tables

**Table 1:** Correlational, descriptive and structural statistics of the UWES-3 items.

	Association and Scalability			Frequency of Responses for the Options						
	Uwes1	Uwes2	Uwes3	1	2	3	4	5	6	7
Uwes1		0.90	0.79	0	5	23	31	61	63	18
Uwes2	0.87		0.87	0	8	20	29	57	52	35
Uwes3	0.77	0.85		1	11	17	31	55	56	30
External criteria										
Work sat.	0.57 ^a^	0.60 ^a^	0.52 ^a^	-	-	-	-	-	-	-
SOS-S	−0.60 ^a^	−0.59 ^a^	−0.59 ^a^	-	-	-	-	-	-	-
GCE	0.33 ^a^	0.35 ^a^	0.36 ^a^	-	-	-	-	-	-	-
Minor acc.	−0.27 ^a^	−0.22 ^a^	−0.21 ^a^	-	-	-	-	-	-	-
Descriptive statistics										
M	5.03	5.14	5.07	-	-	-	-	-	-	-
SD	10.23	10.35	10.38	-	-	-	-	-	-	-
Sk	−0.46	−0.46	−0.51	-	-	-	-	-	-	-
Ku	−0.44	−0.51	−0.43	-	-	-	-	-	-	-
AD	7.19	5.87	6.06	-	-	-	-	-	-	-

Note. All correlations were statistically significant at 0.05 or 0.01. Below the diagonal: Pearson interitem correlations. Above the diagonal: interitem H-scalability coefficients (H_ij_). Work sat.: work satisfaction. SOS-S: stress overload. GCE: general coping effectiveness. Minor acc.: number of minor accidents at work. Sk and Ku: skewness and kurtosis coefficients, respectively. AD: Anderson–Darling normality test. ^a^ Correlations disattenuated by granularity.

**Table 2:** Nonparametric modelling: Mokken scale analysis for the UWES-3.

	H (se)	Monotone Homogeneity		Manifest Invariant Item Ordering (IIO)		r_itc_	r_ps_
		#v	Crit	#v	Crit		
Uwes1	0.85 (0.02)	0	0	0	0	0.85	0.93
Uwes2	0.88 (0.02)	0	0	0	0	0.91	0.96
Uwes3	0.83 (0.03)	0	0	0	0	0.84	0.93
Total	0.85 (0.02)	-	-	-	-	-	-

Note. H: scalability coefficients. se: standard error. #v: number of violations. Crit: weighted criterion. r_itc_: corrected item-test correlation. r_ps_: polyserial correlation.

**Table 3:** Results of ordinal (predictor: UWES-3) and linear (criterion: UWES-3) regression.

	Ordinal Regression (Predictor: UWES-3)		Linear Regression (Criteria: UWES-3)	
	Minor Acc.	Work Sat.	Model 1	Model 2
*R* ^2^ *_McFadden_*	0.03	0.16		
LR test	11.73	20.69 **		
Sex				
Beta	−0.45	−0.34		
OR	0.63	0.7		
Age				
Beta	0.0	0.02		
OR	0.99	1.02		
UWES-3				
Beta	−0.12 **	0.34 **		
OR	0.88	1.41		
*R* ^2^	-	-	0.4	0.11
F (3.197)		-	44.65 **	8.78 **
Sex	-	-		
Beta	-	-	0.02	−0.01
Age	-	-		
Beta	-	-	0.01	0.0
SOS-S	-	-		
Beta	-	-	−0.138 **	
(Beta)^2^	-	-	0.76 **	
GCE	-	-		
Beta	-	-		0.34 **

Note. Work Sat.: work satisfaction. Minor Acc.: minor accidents at work. LR test: likelihood ratio test. OR: odd ratio. SOS-S: stress overload. GCE: general coping effectiveness. ** *p* < 0.001.

## Data Availability

The raw data supporting the conclusions of this article will be made available by the authors, without undue reservation.
